# Progressively Disrupted Brain Functional Connectivity Network in Subcortical Ischemic Vascular Cognitive Impairment Patients

**DOI:** 10.3389/fneur.2018.00094

**Published:** 2018-02-26

**Authors:** Linqiong Sang, Lin Chen, Li Wang, Jingna Zhang, Ye Zhang, Pengyue Li, Chuanming Li, Mingguo Qiu

**Affiliations:** ^1^Department of Medical Imaging, College of Biomedical Engineering, Third Military Medical University, Chongqing, China; ^2^Department of Psychology, Third Military Medical University, Chongqing, China; ^3^Department of Radiology, Southwest Hospital, Third Military Medical University, Chongqing, China

**Keywords:** subcortical ischemic vascular cognitive impairment, graph theoretical analysis, topological organization, network-based statistics, functional connections

## Abstract

Cognitive impairment caused by subcortical ischemic vascular disease (SIVD) has been elucidated by many neuroimaging studies. However, little is known regarding the changes in brain functional connectivity networks in relation to the severity of cognitive impairment in SIVD. In the present study, 20 subcortical ischemic vascular cognitive impairment no dementia patients (SIVCIND) and 20 dementia patients (SIVaD) were enrolled; additionally, 19 normal controls were recruited. Each participant underwent a resting-state functional MRI scan. Whole-brain functional networks were analyzed with graph theory and network-based statistics (NBS) to study the functional organization of networks and find alterations in functional connectivity among brain regions. After adjustments for age, gender, and duration of formal education, there were significant group differences for two network functional organization indices, global efficiency and local efficiency, which decreased (NC > SIVCIND > SIVaD) as cognitive impairment worsened. Between-group differences in functional connectivity (NBS corrected, *p* < 0.01) mainly involved the orbitofrontal, parietal, and temporal cortices, as well as the basal ganglia. The brain connectivity network was progressively disrupted as cognitive impairment worsened, with an increased number of decreased connections between brain regions. We also observed more reductions in nodal efficiency in the prefrontal and temporal cortices for SIVaD than for SIVCIND. These findings indicated a progressively disrupted pattern of the brain functional connectivity network with increased cognitive impairment and showed promise for the development of reliable biomarkers of network metric changes related to cognitive impairment caused by SIVD.

## Introduction

Subcortical ischemic vascular disease (SIVD), characterized by lacunar infarcts and deep white matter changes, frequently causes cognitive impairment in elderly people (1, 2). The cognitive impairment caused by SIVD is classified into the following categories: cognitive impairment no dementia (SIVCIND) and dementia (SIVaD). The predominant cognitive symptoms include dysfunction in attention, information processing, and executive function, probably resulting from ischemic interruption of parallel circuits from the prefrontal cortex to the basal ganglia (1, 3).

Previous studies have identified aberrant changes in both gray matter and white matter in patients with cognitive impairment caused by SIVD. For instance, Thong et al. demonstrated that SIVCIND patients had widespread volume atrophy, especially in the regions of the frontal cortex and subcortical areas (4). Moreover, Seo et al. found cortical thinning in many regions, such as the orbitofrontal cortex and superior temporal gyrus in SIVCIND; in addition to these regions, the dorsolateral prefrontal and temporal cortices were involved in SIVaD (5). Furthermore, Fu et al. observed lower fractional anisotropy values in the fronto-occipital fascicles, genu of the corpus callosum, splenium of the corpus callosum, and superior longitudinal fasciculus in SIVaD by diffusion tensor imaging (6). Lin et al. found decreased fractional anisotropy in some projection fibers and association fibers, such as the posterior thalamic radiation, cingulum, and fronto-occipital fasciculus in SIVCIND (7). Apart from structural studies, several researchers performed resting-state functional magnetic resonance imaging (rs-fMRI) to explore the spontaneous neural activity in cognitive impairment patients with SIVD. Yi et al. observed significant functional alterations in low-frequency oscillation amplitudes in the default-mode network (DMN) in SIVCIND (8). Zhou et al. found decreased functional connectivity in SIVCIND, such as between the thalamus and orbitofrontal lobe and between the medial prefrontal cortex and anterior cingulate cortex (9). Despite increasing knowledge of structural and functional abnormalities in specific brain regions or connections, the exact pathogenesis of cognitive impairment caused by SIVD remains poorly understood.

Recently, graph theoretical approaches have been widely used to reveal many important topological organizations of brain networks, such as network efficiency (10–12). Moreover, disrupted topological organization of whole-brain functional networks has been found in various neurological and psychiatric disorders (13–15). Notably, Yu et al. examined the topological organization of the functional brain network and found decreased global efficiency and local efficiency of the functional network in patients with cognitive impairment caused by SIVD (16). The study by Yu et al. focused on changes in topological organization in patients with cognitive impairment caused by SIVD compared with normal controls; however, very little is known concerning the changes in the topological organization of functional brain networks in relation to the severity of cognitive impairment caused by SIVD or the differences in topological organization among SIVCIND, SIVaD, and normal controls.

The purpose of this study was to investigate changes in the whole-brain functional connectivity network in patients with different levels of cognitive deficits caused by SIVD and explore the relation between changes in brain functional organization and cognitive decline. Here, we hypothesized that the changes in the brain functional connectivity network would progressively increase as cognitive impairment worsened. More specifically, we expected that the progression of cognitive decline would be associated with decreased functional segregation and functional integration of the network. We also hypothesized that the pattern of functional connectivity between brain regions would be progressively disrupted with cognitive decline. Finally, we predicted that network topological properties would be correlated with performance on neuropsychological tests; we conducted this study to determine whether specific changes in the topological organization of the brain are biomarkers of cognitive decline caused by SIVD.

## Materials and Methods

### Participants

A total of 59 right-handed participants, including 20 SIVCIND patients, 20 SIVaD patients, and 19 healthy controls, were recruited. All subjects provided written informed consent in accordance with the Declaration of Helsinki. This study was approved by the local Medical Ethics Committee at the Third Military University (Chongqing, China) on Human Studies.

All of the patients had subcortical WM hyperintensity on T2-weighted imaging with at least two lacunar infarcts, in accordance with the criteria suggested by Galluzzi et al. (17). However, the subcortical WM hyperintensity was not quantified. The Patients with SIVCIND were diagnosed based on the following criteria (18, 19): (1) subjective cognitive complaints reported by the participant or his/her caregiver; (2) insufficient cognitive impairment to meet the DSM-V criteria for dementia; and (3) neuropsychological tests including Hachinski Ischemic Score (HIS) determination (HIS ≥ 7). SIVaD patients were diagnosed on the basis of both DSM-V criteria and the National Institute of Neurological Disorders and Stroke–Association Internationale pour la Recherche et l’Enseignement en Neurosciences (NINDS-AIREN) criteria (20). Exclusion criteria included aphasia and inability to complete psychological testing because of a language disorder, a diagnosed chronic or degenerative disease or condition before stroke affecting the central nervous system, active substance abuse disorders, intracranial hemorrhage and cortical infarct, or epilepsy. However, the exclusion criterion was not including past or present medication for the treatment of cognitive impairment and infarction. Clinical performance was evaluated through the following neuropsychological tasks: Mini-Mental State Examination (MMSE), Clinical Dementia Rating (CDR), Global Deterioration Scale (GDS), Activities of Daily Living Scale (ADL), Montreal Cognitive Assessment (MoCA), and HIS (21–27). All patients received complete sociodemographic and clinical data collection, and two experienced neurologists performed the diagnoses for the two groups.

Nineteen age- and gender-matched healthy volunteers with normal cognitive performance and no known nervous system diseases were recruited as the healthy controls. All healthy controls underwent neurological and neuropsychological evaluation and conventional MRI. None of them had vascular risk factors, cognitive complaints, current psychiatric illness, or a history of psychiatric illness. No healthy controls had brain trauma, brain tumor, psychiatric disorders, systemic disease, or other MRI contraindications such as claustrophobia.

### Data Acquisition

All images were acquired on a 3 T scanner (Magnetom Trio; Siemens Medical Systems, Erlangen, Germany) equipped with eight-channel, phase-array head coils. Foam padding was used to minimize head motion by all subjects. Conventional MRI included transverse T1-weighted images (TR/TE = 200/2.78 ms, flip angle = 70^◦^, matrix = 384 × 384, thickness = 4.0 mm, 25 slices, voxel size = 0.7 mm × 0.6 mm × 5 mm) and fluid-attenuated inversion recovery (TR/TE/TI = 9,000/93/2,500 ms, flip angle = 130°, matrix = 256 × 256, thickness = 4.0 mm, 25 slices, voxel size = 0.9 mm × 0.9 mm × 4 mm). Anatomical image datasets were acquired with a magnetization-prepared rapid gradient echo sequence (TR/TE/TI = 1, 900/2.52/900 ms, flip angle = 9◦, matrix = 256 × 256, thickness = 1.0 mm, no gap, 176 slices, voxel size = 1 mm × 1 mm × 1 mm). Resting-state images were acquired in the axial orientation using a single-shot, gradient-recalled echo planar imaging (EPI) sequence using the following parameters: TR = 2,000 ms, TE = 30 ms, flip angle = 90°, matrix size = 64 × 64, FOV = 192 mm × 192 mm, 36 transverse slices, 3-mm slice thickness without a gap, and resolution = 3 mm × 3 mm × 3 mm. During EPI data acquisition, the subjects were instructed to remain awake, relax and keep their eyes closed without thinking of anything in particular. For each subject, we collected 240 volumes in total.

### Data Preprocessing

Data preprocessing was performed using Statistical Parametric Mapping software (SPM8, http://www.fil.ion.ucl.ac.uk/spm) and Data Processing Assistant for Resting-State fMRI (DPARSF, http://www.restfmri.net). We discarded the first ten volumes to ensure steady-state longitudinal magnetization, and the remaining 230 volumes were corrected for delay in slice acquisition. The images were later coregistered to the first image for correction of rigid-body head motion. For all subjects, excessive motion was defined as more than ±2 mm and ±2° of the translational and rotational parameters. Subsequently, the individual three-dimensional T1-weighted images were coregistered to the mean functional images, which were generated by SPM in the preprocessing step of alignment, after motion correction using a linear transformation. Additionally, we employed multiple linear regression to remove the signal from white matter and cerebrospinal fluid (28, 29). Then, the functional scans were spatially normalized to the standard Montreal Neurological Institute space and resampled to 3 mm isotropic voxels using an optimum 6-parameter affine transformation and nonlinear deformations. As previously suggested, we did not apply spatial smoothing to avoid introducing artificial local spatial correlations (30, 31). Finally, a band-pass filter (0.01–0.08 Hz) was performed to reduce low-frequency drift and high-frequency physiological noise in each voxel (32).

### Network Construction

The nodes and edges between nodes are the two components of a network. In the functional brain network, the nodes represent brain regions. In our study, to determine the nodes of the functional brain network, we used an automated anatomical labeling (AAL) atlas (33) to segment the brain into 90 regions of interest (ROIs) (45 for each hemisphere; details are provided in Table S1 in Supplementary Material). Herein, the functional network was obtained from the defined ROIs and BOLD signal. The nodes represented the 90 ROIs, and the edges represented the Pearson correlation between the mean BOLD time series in two given ROIs. For each participant, a 90 × 90 symmetric weighted network was constructed. Fisher’s *r*-to-*z* transformation was applied to the correlation matrix of each subject to improve the normality of the correlation coefficients (34). Finally, as age, gender, and formal education duration differed across groups, the weighted network was corrected for all three by regressing out these factors from the weighted edges of each participant.

The corrected weighted network was thresholded at different levels of sparsity ranging from 10 to 40% using increments of 1%, keeping the highest weighted edges. At each threshold, network metrics were calculated. The functional organization indices of brain networks were computed using the GRETNA toolbox (https://www.nitrc.org/projects/gretna/).

### Network Analysis

To characterize the weighted brain functional network, several global network parameters were calculated, including the clustering coefficient (*C*_p_), mean path length (*L*_p_), global efficiency (*E*_glob_), and local efficiency (*E*_loc_). The definitions of these metrics are briefly described below.

The clustering coefficient *C*_p_ of a network G of N nodes describes the connectedness of direct neighbors around individual nodes (34). The clustering coefficient *C*_p_ is expressed as follows (35):

$$C_{\text{p}}(G)=\underset{i\in \text{node}}{\text{mean}}\frac{2}{k_{i}(k_{i}-1)}{\sum_{j,k}\left(\bar{w_{ij}}\bar{w_{jk}}\bar{w_{ki}}\right)}^{1/3},$$

where *k_i_* is the degree of node *i*, $\left(\sum_{j\neq i\in G}w_{ij}>0\right)$, and *w* is the weighted value of the edge, which is scaled by the mean of all weights to control each participant’s cost at the same level.

The mean path length *L*_p_ of *G* quantifies the ability to propagate information in parallel. The mean path length *L*_p_ is expressed as follows:

$$L_{\text{p}}(G)=\frac{1}{N(N-1)}\sum_{i\neq j\in G}L_{ij},$$

where *L_ij_* is the shortest path length between any pair of nodes *i* and *j* and is defined as the sum of the edge weight *w_ij_* along this path.

The *E*_g_ of *G* quantifies the global efficiency of parallel information transfer in the network. The *E*_g_ of *G* is expressed as follows:

$$E_{\text{g}}(G)=\frac{1}{N}\sum_{i\in G}E_{\text{nodal\_glob}}(i),$$

where

$$E_{\text{nodal\_glob}}(i)=\frac{1}{N-1}\sum_{i\neq j\in G}\frac{1}{L_{ij}},$$

where *L_ij_* is the shortest path length from node *i* to *j* and is defined as the sum of the edge weight *w_ij_* along this path.

The *E*_loc_ of *G* quantifies the fault tolerance of a network, indicating the capacity for information exchange within each subgraph when the index node is eliminated (30). The *E*_loc_ of *G* is expressed as follows:

$$E_{\text{loc}}(G)=\frac{1}{N}\sum_{i\in G}E_{\text{nodal\_loc}}(i),$$

where

$$E_{\text{nodal\_loc}}(i)=E_{g}(G_{i}),$$

where *G_i_* denotes the subgraph composed of the nearest neighbors of node *i*.

The nodal global efficiency *E*_nodal_glob_ and nodal local efficiency *E*_nodal_loc_ measure the capacity of information propagation of the given node with all other nodes in network and their direct neighbors, respectively.

Furthermore, we calculated the area under the curve (AUC) for each network metric (*C*_p_, *L*_p_, *E*_glob_, *E*_loc_, *E*_nodal_glob_, and *E*_nodal_loc_) to provide a summarized scalar for the topological characterization of brain networks independent of single threshold selection (36).

To determine whether a combination of graph-theory measures could predict the subject groups, we performed Adaptive Boosting, a machine learning meta-algorithm used to build classification models. We chose a decision tree as a weak learner, and the number of learning cycles was 100. In our model, the predictors were the four global network metrics (*C*_p_, *L*_p_, *E*_g_, and *E*_loc_), and the response was the subject’s group. Classification accuracy was evaluated *via* leave-one-out cross-validation to ensure a relatively unbiased estimate of the generalization power of the classifiers to new subjects. The sensitivity and the specificity were calculated from the confusion matrix.

To further identify functional connections showing differences in patient groups, we used the network-based statistics (NBS) approach, which is a validated, nonparametric statistical approach for controlling family-wise error in connectome analyses (37). NBS was applied to compare the patient and normal control groups with the following sequence: (1) one-way ANOVA (*post hoc*: two-sample one-tailed *t*-tests) was performed to compare the strength of the edge weight at each individual element of the connectivity matrix, (2) network components of connected edges that survived a *p*-value of 0.005 uncorrected were retained, and (3) the size of the largest cluster was calculated. To generate an empirical null distribution for evaluating the statistical significance of the cluster sizes, we randomly shuffled the groups (10,000 permutations) and obtained the largest cluster size null distribution by repeating steps 1, 2, and 3. Finally, for any connected component of size *M* that found in the right grouping of controls and patients, the corrected *p*-value was determined by calculating the proportion of the 10,000 permutations for which the maximal connected component was larger than *M*.

Corrected weighted networks of all groups were entered into one-way ANOVA with the NBS approach (*p* < 0.01 NBS corrected for multiple comparisons). *Post hoc* two-sample one-tailed *t*-tests were performed to assess between-group differences in the significant network obtained by ANOVA (*p* < 0.01 NBS corrected for multiple comparisons).

For visualization purposes, circular graphical representations were used to display significant connections in the statistical analyses using circos software (http://www.circos.ca) (38). Pair-wise connections were displayed with links colored by connection type as follows: left intrahemispheric (blue), right intrahemispheric (green), and interhemispheric (red). ROIs were grouped according to Salvador (i.e., frontal, temporal, parietal, medial temporal, occipital, and subcortical) (39). The ROIs with a high degree of significant connections (*k* > 1 SD above the mean) were classified as “network hubs” and represented in blue, in accordance with a previous study (10).

### Statistical Analysis

All statistical analyses were performed using SPSS Statistics 22.0.0 (http://www-01.ibm.com/software/analytics/spss/).Pearson’s chi-squared test was used to compare sex categorical variables. Three-level one-way ANOVAs were used to compare clinical and sociodemographic data between the NC and two patient subgroups. Two-sample *t*-test was used to detect differences in clinical scale scores (HIS, GDS, CDR, and ADL) between SIVCIND and SIVaD patients. The statistical significance threshold was set at *p* < 0.05.

Between-group differences in AUC values of global network metrics (*C*_p_, *L*_p_, *E*_g_, and *E*_loc_) and nodal efficiency (*E*_nodal_glob_ and *E*_nodal_loc_) were investigated with one-way ANOVA after the normality of the data distribution was assessed with a Kolmogorov–Smirnov test. Levene’s test showed nonhomogeneity of variances within groups; thus, Welch ANOVA was performed on the data. Metrics showing a main effect of group differences in the ANOVA model were further evaluated by *post hoc* tests (Games-Howell test). A significance threshold of *p* < 0.05 was applied to each test, and the FDR was used to correct for multiple comparisons.

In addition, to explore the relationships between topological properties of the network measures and clinical outcomes in cognitive function, we further performed Spearman’s correlation between topological properties (*C*_p_, *L*_p_, *E*_g_, and *E*_loc_) and cognitive test scores (MMSE and MoCA) in patient groups containing both SIVCIND and SIVaD patients. *Z*-scores were calculated for each of these cognitive scores. We used a statistical significance level of *p* < 0.05, and the FDR was used to correct for multiple comparisons.

## Results

### Clinical Statistics

Subject demographics are listed in Table 1. Significant differences between the three groups (NC, SIVCIND, and SIVaD) were found in MMSE (*F* = 93.322, *p* < 0.001) and MoCA scores (*F* = 115.981, *p* < 0.001). Games-Howell tests revealed that SIVaD patients and SIVCIND patients showed lower MMSE and MoCA scores than did normal controls, and SIVaD patients showed lower scores than did SIVCIND patients. In addition, two-sample *t*-test revealed that SIVCIND patients showed lower HIS (*T* = −3.498, *p* = 0.001), GDS (*T* = −5.121, *p* < 0.001), and CDR (*T* = −6.658, *p* < 0.001) scores than did SIVaD patients. There was no significant difference among the three groups in gender (χ^2^ = 0.923, *p* = 0.632), age (*F* = 0.663, *p* = 0.519), and duration of formal education (*F* = 1.695, *p* = 0.193).

**Table 1:** Demographics and clinical characteristics of the subjects.

	NC (*n* = 19)	SIVCIND (*n* = 20)	SIVaD (*n* = 20)	*p*-Value
Gender (male/female)	9/10	8/12	11/9	0.519^a^
Age (years)	58–78 (67.7 ± 5.2)	47–82 (65.2 ± 9.2)	57–85 (67.1 ± 7.2)	0.663^b^
Education (years)	3–15 (9.1 ± 3.8)	1–12 (7.7 ± 3.3)	0–15 (6.9 ± 3.6)	0.179^b^
MMSE	27–30 (28.5 ± 0.8)	22–28 (24.1 ± 1.9)	4–22 (14.2 ± 5.4)	<0.001^b^
MoCA	27–30 (28.0 ± 0.9)	8–24 (17.9 ± 4.9)	1–20 (8.9 ± 4.5)	<0.001^b^
HIS	–	7–12 (8.5 ± 1.8)	8–17 (8.9 ± 4.5)	0.001^c^
GDS	–	2–5 (3.3 ± 0.7)	4–7 (4.7 ± 1.0)	<0.001^c^
CDR	–	0–0.5 (0.5 ± 0.2)	1–3 (1.5 ± 0.7)	<0.001^c^
ADL	–	12–25 (15 ± 3.2)	12–19 (14.4 ± 2.5)	0.481^c^

*Data are expressed as the range from min–max (mean ± SD)*.

*^a^The p-value was obtained using Pearson’s Chi-squared test*.

*^b^The p-value was obtained using three levels one-way ANOVA*.

*^c^The p-value was obtained using two-sample t-test*.

*MMSE, Mini-Mental State Examination; MoCA, Montreal Cognitive Assessment; HIS, Hachinski Ischemic Score; GDS, Global Deterioration Scale; CDR, Clinical Dementia Rating; ADL, Activities of Daily Living Scale*.

### Altered Global Topological Properties

**Figure 1:**
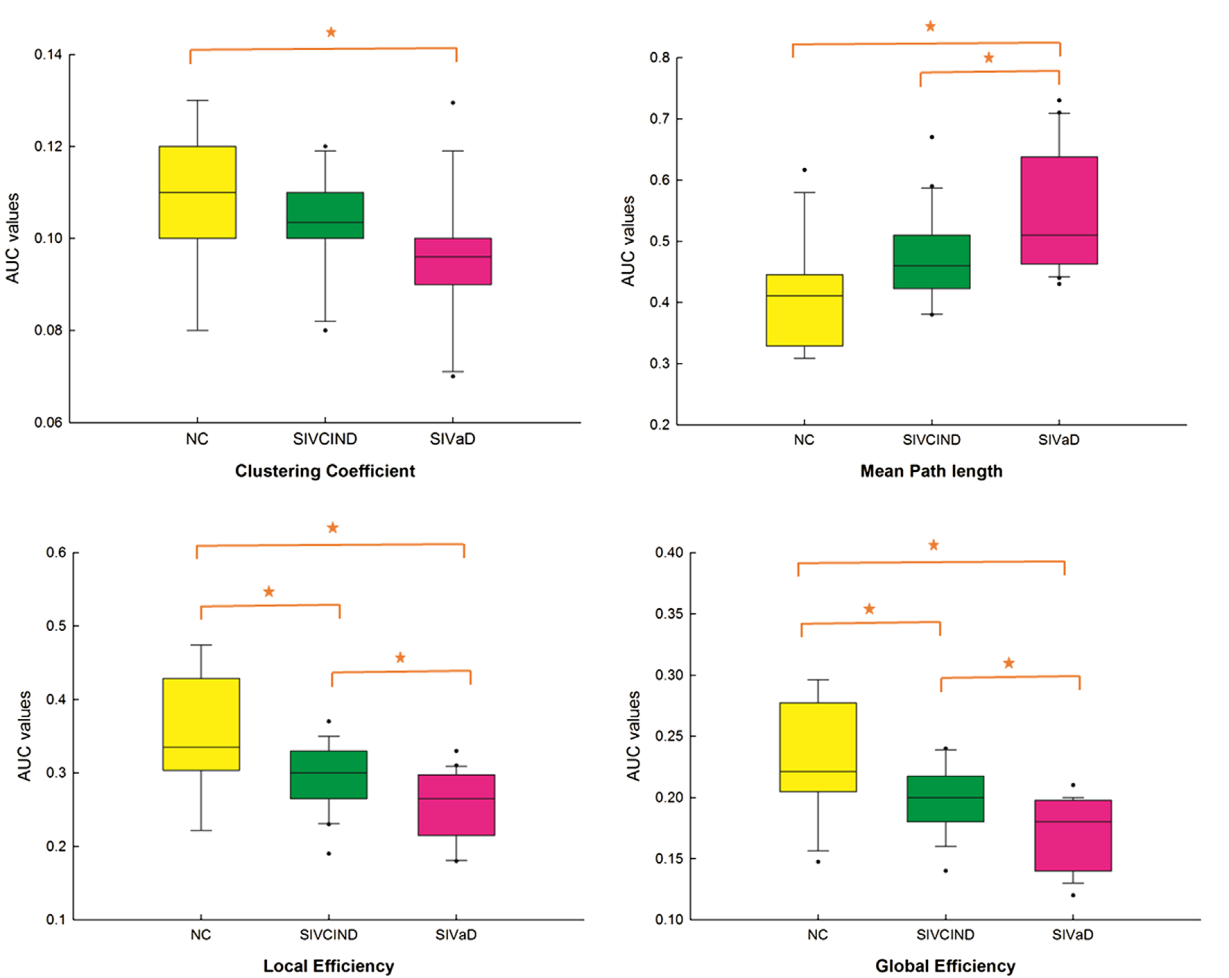
Boxplot of mean area under the curve (AUC) network metric values for each group. Between-group differences were assessed by one-way ANOVA and *post hoc* Games-Howell test (*p* < 0.05). The AUC value corresponds to the AUC in plotting the network metric values as a function of the sparsity applied to the connectivity matrix.

There was a significant difference between the groups as determined by one-way ANOVA for the clustering coefficient *C*_p_ (*F* = 3.761, *p* = 0.033), mean path length *L*_p_ (*F* = 9.042, *p* = 0.001), global efficiency *E*_g_ (*F* = 11.069, *p* < 0.001), and local efficiency *E*_loc_ (*F* = 10.472, *p* < 0.001) (Figure 1). A Games-Howell test revealed that *E*_g_ and *E*_loc_ were significantly lower for SIVCIND and SIVaD than for NC. Additionally, SIVaD showed lower *E*_g_ and *E*_loc_ values than did SIVCIND. The *C*_p_ values were significantly higher for NC than for SIVaD. The *L*_p_ values were significantly higher for SIVaD than for SIVCIND and NC.

### Between-Group Differences in Nodal Efficiency

**Figure 2:**
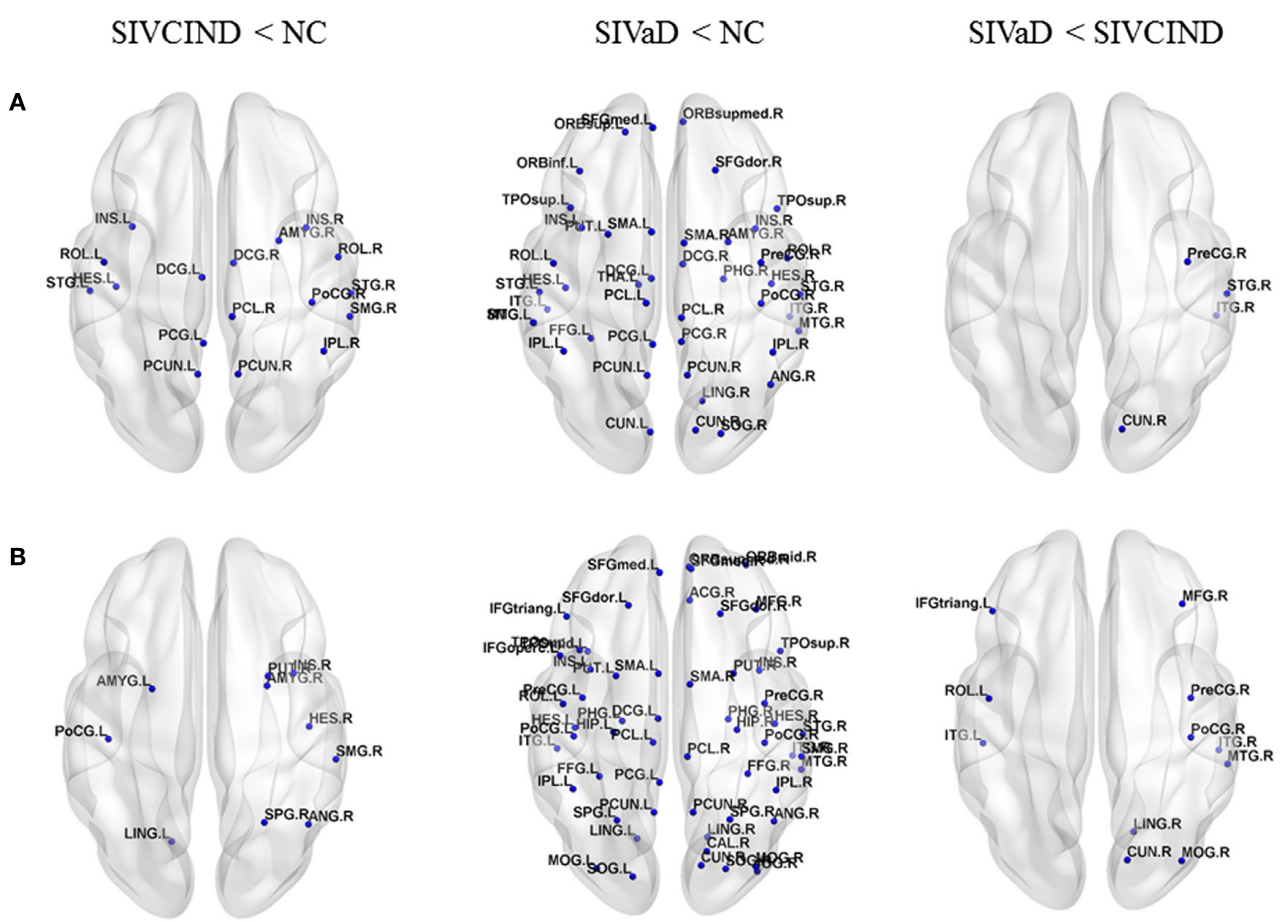
Brain regions showing significant differences in nodal efficiency among SIVCIND, SIVaD, and normal controls. The results were visualized using BrainNet Viewer (NKLCNL, Beijing Normal University). Three-dimensional maps show significant differences in **(A)** nodal global efficiency and **(B)** nodal local efficiency among SIVCIND, SIVaD, and normal controls. Detailed brain region information corresponding to the anatomical labels can be found in Table S1 in Supplementary Material.

Compared with NC, SIVCIND patients showed decreased nodal global efficiency in several regions located in the parietal and temporal cortices (Figure 2A). In addition to these regions, SIVaD showed decreased nodal global efficiency in the prefrontal and occipital cortices and more temporal areas (Figure 2A). Moreover, compared with SIVCIND patients, SIVaD patients exhibited lower nodal global efficiency in the right precentral gyrus, cuneus, superior temporal gyrus, and inferior temporal gyrus (Figure 2A).

Additionally, compared with NC, SIVCIND patients showed decreased nodal local efficiency predominantly in the right putamen, superior parietal gyrus, supramarginal gyrus, angular gyrus, and bilateral amygdala (Figure 2B). SIVaD had widely decreased nodal local efficiency in the whole brain (Figure 2B). Compared with SIVaD patients, SIVCIND patients had higher nodal local efficiency in the right precentral and postcentral gyrus and some default-mode regions (Figure 2B). Compared with NC, patients did not show a significant increase in nodal global efficiency and nodal local efficiency.

### Altered Functional Connectivity

**Figure 3:**
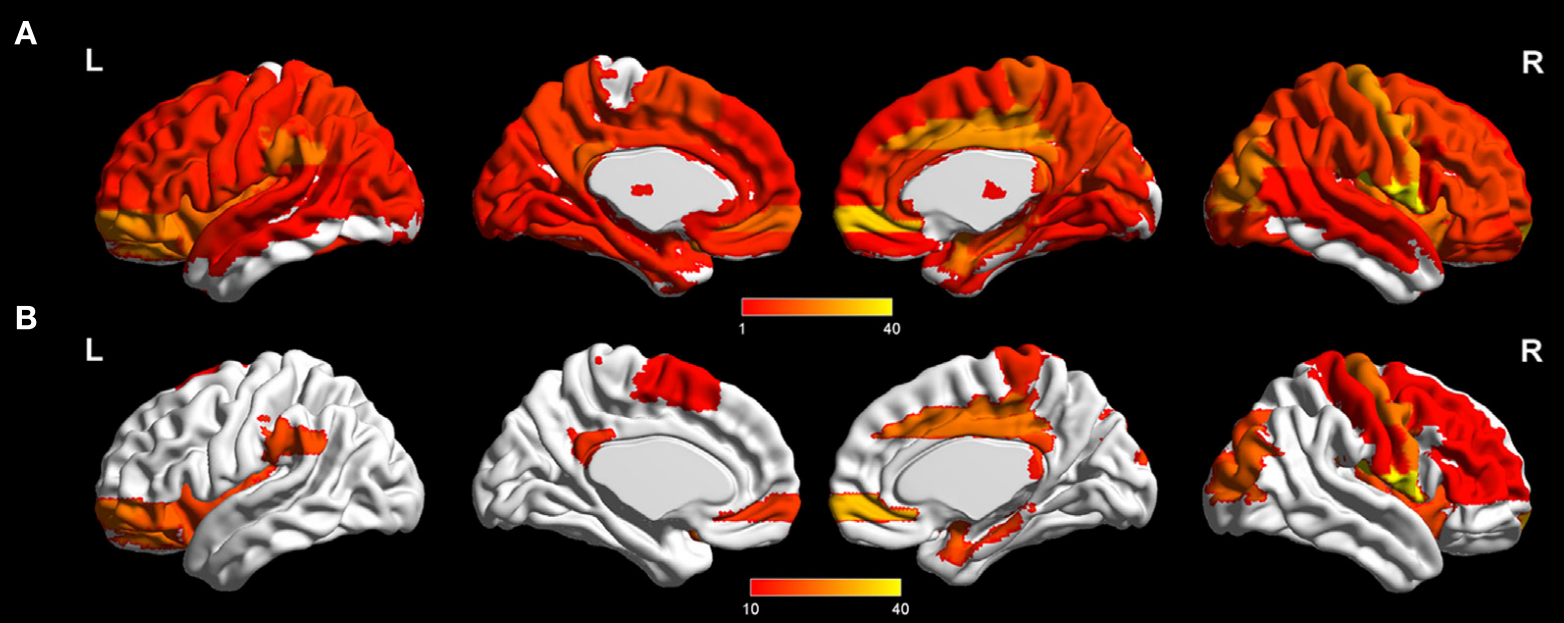
Significant difference in terms of functional connections between groups as determined by one-way ANOVA (network-based statistics corrected, *p* < 0.01). **(A)** Significant brain network nodes are rendered on the surface of automated anatomical labeling atlas in BrainNet Viewer (NKLCNL, Beijing Normal University). The color code represents the number of significant connections of the given regions of interest. **(B)** Significant cortical and subcortical nodes with a number of significant connections higher than 10 are displayed.

**Figure 4:**
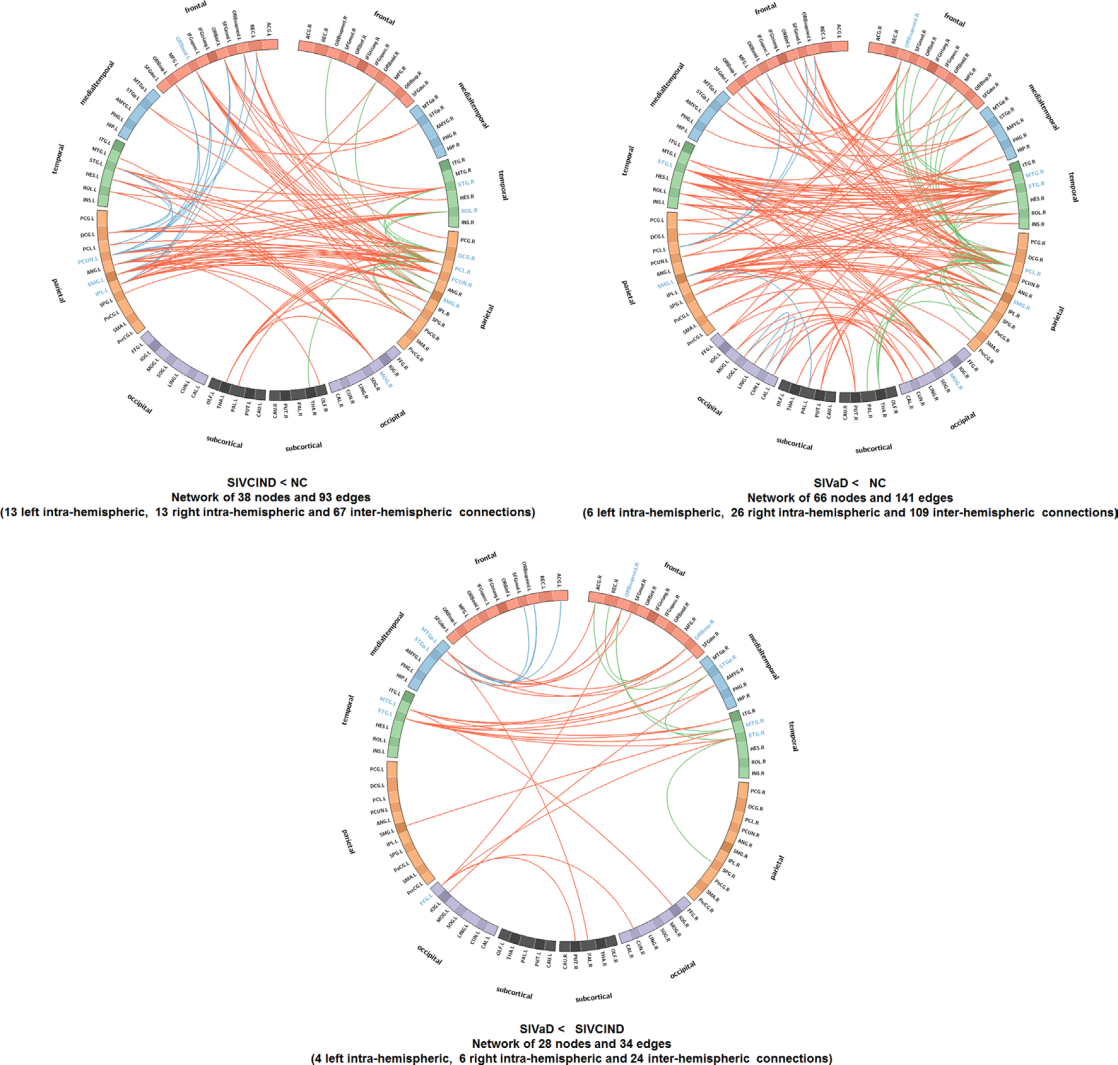
Connectograms show altered functional connections among SIVCIND, SIVaD, and NC (NBS corrected, *p* < 0.01). Links are colored by connection type as follows: left intrahemispheric (blue), interhemispheric (red), and right intrahemispheric (green). ROIs were grouped according to Salvador et al. (39) (i.e., frontal, temporal, parietal, medial temporal, occipital, and subcortical). The ROIs with a high degree of significant connections (*k* > 1 SD above the mean) are presented in blue.

ANOVA revealed significant functional differences between groups (NBS corrected, *p* < 0.01) in almost the whole brain (Figure 3; Tables 2 and 3). *Post hoc* analysis revealed between-group differences in the architectural features of connections and hub nodes; these differences are illustrated by connectograms (Figure 4; Tables 2 and 3). Compared with NC, SIVCIND showed 93 significantly decreased connections, and SIVaD exhibited 141 significantly decreased connections. Notably, the temporal and medial temporal cortices played more important roles in significantly decreased connections in SIVaD (39%) than in SIVCIND (26.8%). Additionally, compared with SIVCIND, SIVaD exhibited 34 significantly decreased connections, which were mostly located in the frontal/temporal and temporal/temporal cortices.

**Table 2:** Significant group differences (ANOVA, *p* < 0.01 NBS corrected for multiple comparisons).

Brain area	Side	Regions
Frontal	Right	Superior frontal gyrus, medial orbital
		Superior frontal gyrus, dorsolateral part
		Middle frontal gyrus
		Inferior frontal gyrus, triangularis part
	Left	Superior frontal gyrus, orbital part
		Middle frontal gyrus, orbital part
		Superior frontal gyrus, medial orbital
		Inferior frontal gyrus, orbital part

Parietal	Right	Precentral gyrus
		Middle cingulate gyrus
		Postcentral gyrus
		Posterior cingulate gyrus
		Paracentral lobule
	Left	Supramarginal gyrus
		Supplementary motor area
		Posterior cingulate gyrus
		Precuneus

Medial temporal	Right	Amygdala
		Parahippocampus
		Hippocampus

Temporal	Right	Rolandic operculum
		Insula
	Left	Insula

Occipital	Right	Middle occipital gyrus
		Superior occipital gyrus
		Cuneus

Subcortical	Left	Putamen

*Only cortical and subcortical regions with a number of significant connections higher than 10 are reported*.

**Table 3:** Group comparisons (two-sample *t*-test, *p* < 0.01 NBS corrected for multiple comparisons).

Contrast	Side	Brain regions
NC vs. SIVCIND	Right	Paracentral lobule
		Precuneus
		Supramarginal gyrus
		Middle cingulate gyrus
		Rolandic operculum
		Middle occipital gyrus
		Superior temporal gyrus
	Left	Precuneus
		Inferior parietal gyrus
		Middle frontal gyrus, orbital part
		Supramarginal gyrus

NC vs. SIVaD	Right	Paracentral lobule
		Superior temporal gyrus
		Middle temporal gyrus
		Superior frontal gyrus, medial orbital
		Supramarginal gyrus
		Middle occipital gyrus
	Left	Superior temporal gyrus
		Supramarginal gyrus

SIVCIND vs. SIVaD	Right	Superior frontal gyrus, medial orbital
		Superior frontal gyrus, orbital part
		Superior temporal gyrus
		Temporal pole: superior temporal gyrus
	Left	Temporal pole: middle temporal gyrus
		Superior temporal gyrus
		Fusiform gyrus
		Middle temporal gyrus
		Temporal pole: superior temporal gyrus

*Only cortical and subcortical regions with a high degree of significant connections (*k* > 1 SD above the mean) are reported*.

Moreover, compared with NC, SIVCIND had lower hub connections in the precuneus and supramarginal gyri in the bilateral hemisphere; in the middle cingulate gyrus, rolandic operculum, and superior temporal gyrus in the right hemisphere; and in the inferior parietal gyrus and orbital middle frontal gyrus in the left hemisphere. SIVaD had lower hub connections in the superior temporal gyrus and supramarginal gyri in the bilateral hemisphere and in the paracentral lobule, middle temporal gyrus, superior frontal gyrus (medial orbital), and middle occipital gyrus in the right hemisphere. In addition, compared with SIVaD, SIVCIND had higher hub connections in the superior frontal gyrus (medial orbital and orbital areas) and some temporal areas.

### Relationships between Topological Properties and Cognitive Test Scores

**Figure 5:**
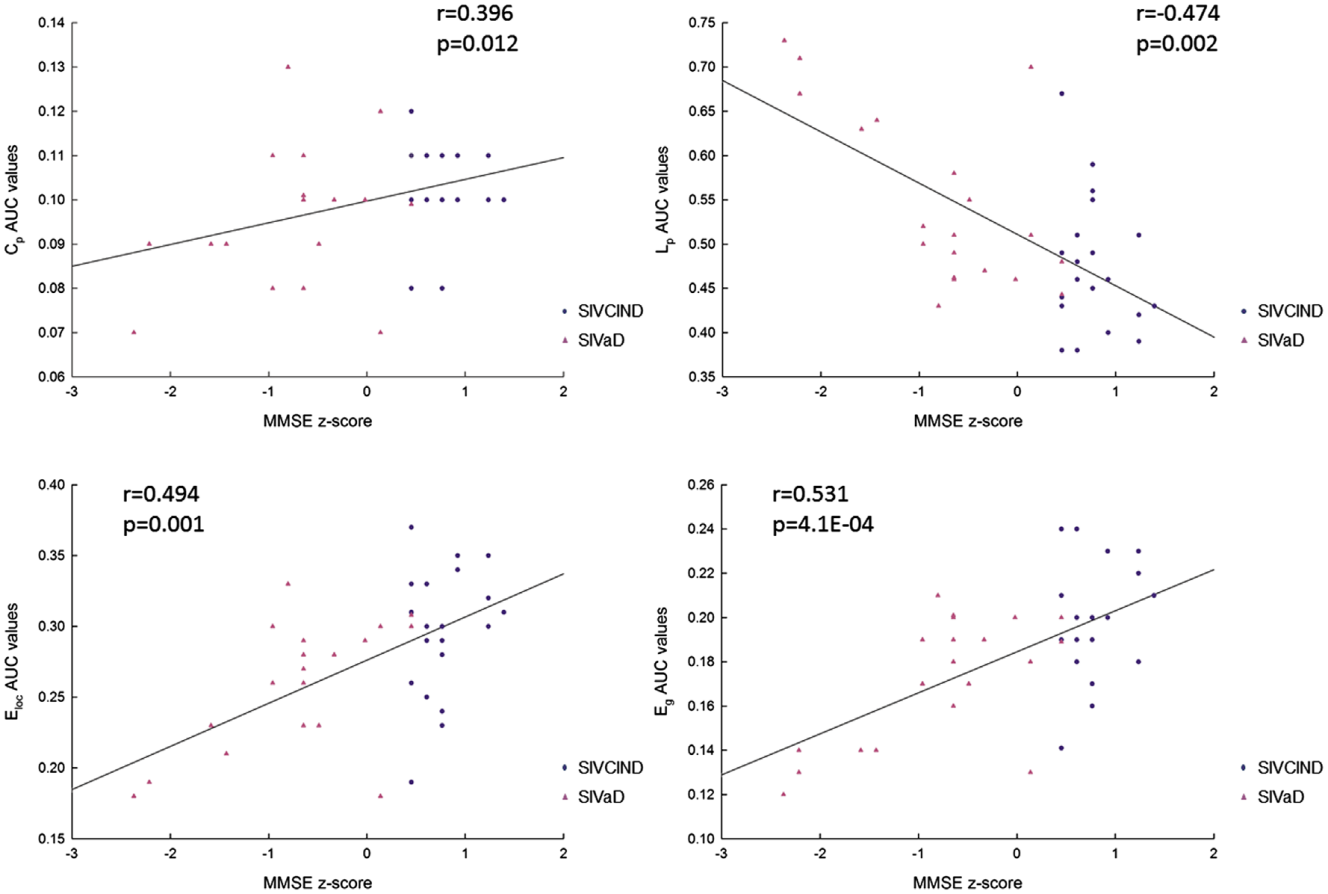
The correlation between each area under the curve (AUC) network metric value and Mini-Mental State Examination (MMSE) scores (*p* < 0.05, FDR corrected). *C*_p_: clustering coefficient; *L*_p_: mean path length; *E*_glob_: global efficiency; and *E*_loc_: local efficiency.

**Figure 6:**
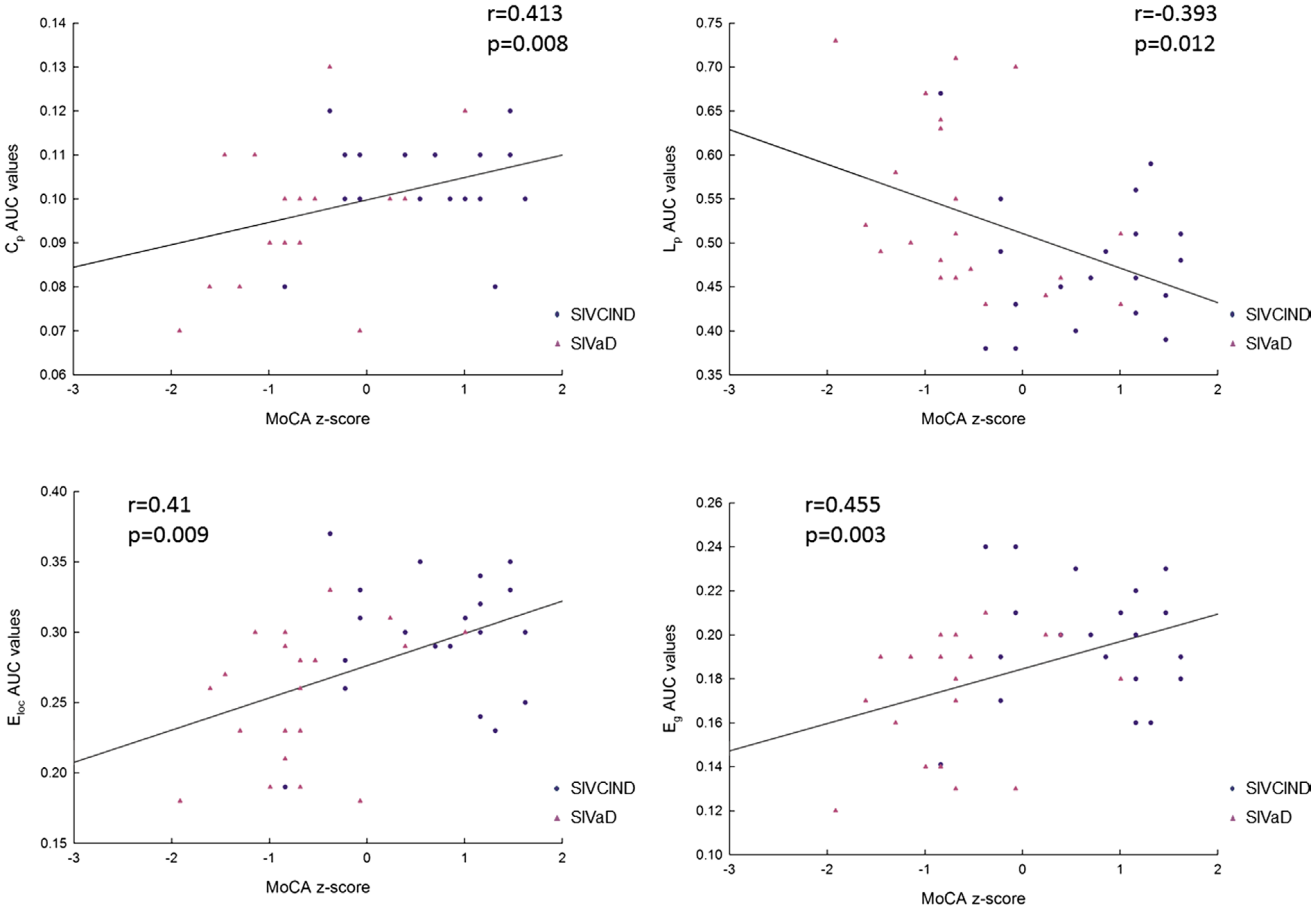
The correlation between each area under the curve (AUC) network metric value and Montreal Cognitive Assessment (MoCA) scores (*p* < 0.05, FDR corrected). *C*_p_: clustering coefficient; *L*_p_: mean path length; *E*_glob_: global efficiency; and *E*_loc_: local efficiency.

Correlation analysis showed that MMSE and MoCA scores were positively correlated with *C*_p_, *E*_loc_, and *E*_g_ values and negatively correlated with *L*_p_ values (Figures 5 and 6).

### Sensitivity and Specificity of Network Properties in Differentiating Patients from Normal Controls

The leave-one-out cross-validation classification procedure was applied using the four network metrics, and the performance of the procedure is summarized in Table 4. A classification accuracy of 76% was obtained for all subjects. However, the accuracy varied between groups. The procedure was good for distinguishing SIVaD patients from normal controls with a sensitivity of 95% and a specificity of 90%. More difficulties were observed when distinguishing SIVCIND patients from normal controls with a sensitivity of 89% and a specificity of 65%.

**Table 4:** Performance of subjects’ classification based on global network metrics.

	SIVCIND	SIVaD
Sensitivity	0.89	0.95
Specificity	0.65	0.90

## Discussion

In this study, we investigated the altered topological properties of the functional network in patients with cognitive impairment caused by SIVD. The results revealed that the local efficiency (measured functional segregation) and global efficiency (measured functional integration) decreased in both SIVCIND and SIVaD patients; more specifically, the clustering coefficient, local efficiency and global efficiency decreased, and the mean path length increased as cognitive impairment worsened. These results implied a disturbance in information exchange in the brain network. Moreover, group differences in functional connectivity mainly concerned the orbital prefrontal, parietal, temporal and subcortical cortices. The brain connectivity network was progressively disrupted as cognitive impairment worsened, with an increased number of decreased connections between brain regions. Additionally, we observed more decreased nodal global and local efficiency in the prefrontal and temporal cortices in SIVaD. These results provided insights into the relationship between altered topological organization and cognitive deficits caused by SIVD.

### Altered Global Topological Properties in SIVCIND and SIVaD Compared with Normal Controls

As shown by the progressive decrease in the values of the clustering coefficient, local efficiency, and global efficiency as cognitive impairment increased, there was a reduction in the functional integration and functional segregation of the brain with cognitive impairment. Functional segregation in the brain represents specialized processing within densely interconnected groups of brain regions, and functional integration in the brain represents the ability to rapidly combine specialized information from distributed brain regions (40). This information suggests that progressive cognitive impairments in SIVD may be related to the progressive decrease in the density of connections involved in specialized networks engaged in cognition and the progressive decrease in long-distance functional connections. Specifically, MMSE and MoCA scores were positively correlated with functional integration and segregation of the brain. Hence, decreased performance on the cognitive tests among patients with cognitive impairment caused by SIVD was associated with less extended local clustering, less efficient communication among neighboring areas, and a reduced ability to combine specialized information from distributed brain regions. This finding is in line with the results of previous studies investigating functional organization. For instance, Yu et al. (16) found that compared with normal controls, subcortical vascular cognitive impairment patients exhibited a decreased clustering coefficient and global efficiency in parietal and temporal areas; these changes were correlated with cognitive test performance. By selecting the thalamus as the seed, Zhou et al. (9) reported that MMSE scores correlated with decreased functional connectivity between the left thalamus and left olfactory bulb in subcortical vascular cognitive impairment patients.

### Decreased Nodal Efficiency in SIVCIND and SIVaD Compared with Normal Controls

In addition to global metrics, we also studied local and global efficiency at a nodal level to show the extent of information transmission capacity of nodes with their neighbors and all other nodes in networks. As shown in Figure 2, the brain regions with altered nodal efficiency progressively extended over the whole brain as cognitive impairment worsened. Compared with NC, SIVCIND and SIVaD did not exhibit significantly increased nodal efficiency, which suggested reduced information transmission between a node and its neighbors and all other nodes in the network in patients with cognitive impairment caused by SIVD. Specifically, we found that SIVCIND showed decreased nodal efficiency in several brain regions involved in the parietal/temporal cortex. When cognitive impairment increased (SIVaD), more brain regions with decreased nodal efficiency were observed in the parietal/temporal lobe. Structural abnormality evidence has shown that the cortex thins in the parietal and temporal cortices as cognitive impairment worsens (4). Thus, this finding is compatible with previous studies, and the gradual progression of decreased nodal efficiency in parietal/temporal areas suggested that their lower capacity for information exchange with other nodes in the network presumably occurred in response to non-cognitive consequences such as apathy and slower motor function in patients with cognitive impairment caused by SIVD (3, 41, 42).

Moreover, SIVCIND had lower nodal global efficiency in the lateral DMN, such as the precuneus and left posterior cingulate gyrus, in line with previous studies (9, 43). When cognitive impairment increased (SIVaD), the regions of significantly decreased nodal global efficiency extended to the prefrontal cortex, including the medial superior frontal gyrus and orbital and dorsolateral frontal cortices, which are the primary components of the prefrontal/subcortical circuit, and the interruption of this circuit was associated with executive dysfunction (1, 3), which is a specific cognitive profile in cognitive deficits affected by vascular lesions (44). Additionally, a similar pattern of progression was observed in the prefrontal cortex with regard to nodal local efficiency as cognitive impairment increased. Thus, the nodal efficiency dysfunction in the prefrontal cortex could be used to determine whether patients are at risk of major cognitive impairment or dementia. Compared with SIVCIND, SIVaD had lower nodal global and local efficiency in the temporal cortex and several occipital areas, such as the cuneus and lingual gyrus; both structural and functional abnormalities in these areas have been associated with depression (14, 45). Depression is particularly prominent in those with vascular dementia (2). Our findings are consistent with these previous studies and suggested decreased information exchange in occipital areas, which presumably occurred in response to depression frequently caused by SIVaD. Unfortunately, the present study included no neuropsychiatric test to estimate the state of neuropsychiatric symptoms such as depression.

### Decreased Functional Connectivity in SIVCIND and SIVaD

The NBS analyses showed that alterations in brain network functional connectivity progressively increased as cognitive impairment worsened (Figure 4). A structural study found that vertices with cortical thinning largely overlapped between SIVaD and SIVCIND, but their extent and severity were greater in SIVaD than in SIVCIND (5, 46). The progressive increase in reduced connectivity with worse cognitive impairment was in accordance with the extent and severity of cortical thinning.

Compared with normal controls, SIVCIND exhibited weakened connections mainly located in the frontal cortex and parietal areas. An examination of the connectogram (Figure 4, top-left) shows that these alterations mainly concerned interhemispheric connections to the frontal and parietal cortices, which were also found in an electroencephalography study (47). Changes in the frontoparietal attentional network (48) agree with the attention deficits usually exhibited by patients with cognitive impairment caused by SIVD. Notably, significantly decreased functional connectivity in the orbitofrontal cortex in SIVCIND is known to be involved in executive function (49, 50). When cognitive deficits worsened (SIVaD), the functional network was widely altered (Figure 4, top-right). Specifically, SIVaD exhibited more decreased connections in the orbitofrontal cortex and dorsolateral prefrontal cortex than did normal controls. The orbitofrontal cortex and dorsolateral prefrontal cortex are the primary components of the prefrontal/subcortical circuit, and the interruption of this circuit was associated with executive dysfunction (1, 3). The progressively increased changes in functional connectivity in the prefrontal cortex were in accordance with the progression in nodal properties and further suggested that the dysfunction of the prefrontal cortex could be used to determine the severity of cognitive decline in SIVD. Additionally, more significantly decreased connections were observed in the temporal and medial temporal cortices for SIVaD than for SIVCIND. Previous studies suggested that the temporal cortex is important in regulating complex social interactions (51) and serves as an important component of the social perception system (52). Therefore, decreased functional connections associated with temporal areas may explain the impaired social behaviors and loss of empathy that are frequently observed in SIVaD. Moreover, the medial temporal cortex is thought to be involved in encoding declarative memory (53), and decreased connections in this area can lead to forgetfulness, a clinical manifestation of SIVaD (3).

Compared with SIVCIND, SIVaD showed weakened connections that mainly concerned interhemispheric connections from the temporal cortex to the temporal, occipital and orbitofrontal cortices (Figure 4, bottom). Additionally, almost all the network hubs were located in temporal areas (Table 3). Particularly, Seo et al. (5) found that SIVaD showed cortical thinning in all these areas involved in SIVCIND in addition to the temporal cortices. Our findings are consistent with previous studies and further suggested that temporal areas may be associated with cognitive decline from SIVCIND to SIVaD.

Several limitations in our study need to be further addressed. First, the functional brain network was constructed at a regional level based on a previously published atlas (AAL) by parcellating the entire brain into 90 regions. Brain networks derived using different parcellation schemes exhibit distinct topological organization (54). In future, different brain parcellation schemes should be used to determine which is most appropriate for characterization of topological architecture in cognitive deficits affected by SIVD. Second, in our study, no neuropsychiatric scale was used to verify the relationship between the abnormal topological properties and neuropsychiatric symptoms observed in patients. However, neuropsychiatric symptoms such as depression are particularly prominent in SIVaD patients. In the future, we will collect detailed data using a neuropsychiatric scale to evaluate the neuropsychiatric impairment associated with subcortical vascular lesions. Third, the sample size in the current study was not large. Although our sample has the important advantage of comprising different levels of cognitive deficits in patients affected by SIVD, our study may have lacked sufficient statistical power to detect some subtle effects among the three groups. Fourth, the number and location of the lesions is not uniform across the patients in our study. In the further studies, more patients will be recruited to ensure the lesions consistently across all patients as far as possible. Finally, similar to most rs-fMRI studies, the interference of certain potential confounding factors, such as respiratory and cardiac cycle artifacts, could not be eliminated by a slow sampling rate, despite using a band-pass filter of 0.01–0.08 Hz.

## Conclusion

Our results revealed that progressive cognitive impairment caused by SIVD is associated with changes in the brain functional connectivity network. The topological organization of the network was progressively disrupted as cognitive impairment worsened, with an increased network mean path and decreased network global efficiency, local efficiency and clustering coefficient. Decreased functional connectivity between brain regions progressively increased as cognitive deficits increased, especially in the prefrontal and temporal cortices. Moreover, between-group differences in nodal properties showed that brain regions with decreased nodal efficiency progressively extended to the prefrontal (orbital and dorsolateral parts) and temporal cortices. These findings suggested a progressively disrupted pattern in the brain functional connectivity network as cognitive impairment increased; these results hold promise for developing reliable biomarkers of network metric changes related to cognitive decline in SIVD.

## Author Contributions

Conceived and designed the experiments: LS, LC, CL, and MQ. Performed the experiments: LS, LW, JZ, YZ, and PL. Analyzed the data: LS, LC, and LW. Contributed reagents/materials/analysis tools: JZ and YZ. Wrote the article: LS. Figures processing: LS and LC.

## Conflict of Interest Statement

The authors declare that the research was conducted in the absence of any commercial or financial relationships that could be construed as a potential conflict of interest.
