# Supplementary material for: Progressively Disrupted Brain Functional Connectivity Network in Subcortical Ischemic Vascular Cognitive Impairment Patients
Source: Front Neurol. 2018 Feb 26;9:94. doi: 10.3389/fneur.2018.00094 (PMC5834750; doi:10.3389/fneur.2018.00094)
Supplement: Supplementary file 1 [file table_1.docx]

Disrupted Brain Functional Connectivity Network in Subcortical Ischaemic Vascular Cognitive Impairment Patients

Linqiong Sang^1#^, Lin Chen^2#^, Li Wang^1^, Jingna Zhang^1^, Ye Zhang^1^, Pengyue Li^1^, Chuanming Li^3*^, Mingguo Qiu^1*^

^1^Department of Medical Imaging, College of Biomedical Engineering, Third Military Medical University, Chongqing, China

^2^ Department of Psychology, Third Military Medical University, Chongqing, China

^3^Department of Radiology, Southwest Hospital, Third Military Medical University, Chongqing, China

^#^These authors contributed equally to this work.

^*^Corresponding authors:

M. Qiu

E-mail address: qiumingguo@yahoo.com

C. Li

E-mail address: li_chuanming@yeah.net

**Supplementary materials**

**Table S1** Anatomical regions of interest (ROIs)

| Index | Regions | Abbr. |
| --- | --- | --- |
| Frontal |  |  |
| 3,4 | Frontal_Sup_L | SFGdor.L |
| 5,6 | Frontal_Sup_Orb_L | ORBsup.L |
| 7,8 | Frontal_Mid_L | MFG.L |
| 9,10 | Frontal_Mid_Orb_L | ORBmid.L |
| 11,12 | Frontal_Inf_Oper_L | IFGoperc.L |
| 13,14 | Frontal_Inf_Tri_L | IFGtriang.L |
| 15,16 | Frontal_Inf_Orb_L | ORBinf.L |
| 23,24 | Frontal_Sup_Medial_L | SFGmed.L |
| 25,26 | Frontal_Mid_Orb_L | ORBsupmed.L |
| 27,28 | Rectus_L | REC.L |
| 31,32 | Cingulum_Ant_L | ACG.L |
| Parietal-(pre)motor |  |  |
| 1,2 | Precentral_L | PreCG.L |
| 19,20 | Supp_Motor_Area_L | SMA.L |
| 33,34 | Cingulum_Mid_L | DCG.L |
| 35,36 | Cingulum_Post_L | PCG.L |
| 57,58 | Postcentral_L | PoCG.L |
| 59,60 | Parietal_Sup_L | SPG.L |
| 61,62 | Parietal_Inf_L | IPL.L |
| 63,64 | SupraMarginal_L | SMG.L |
| 65,65 | Angular_L | ANG.L |
| 67,68 | Precuneus_L | PCUN.L |
| 69,70 | Paracentral_Lobule_L | PCL.L |
| Temporal |  |  |
| 29,30 | Insula_L | INS.L |
| 17,18 | Rolandic_Oper_L | ROL.L |
| 79,80 | Heschl_L | HES.L |
| 81,82 | Temporal_Sup_L | STG.L |
| 85,86 | Temporal_Mid_L | MTG.L |
| 89,90 | Temporal_Inf_L | ITG.L |
| Medial temporal |  |  |
| 37,38 | Hippocampus_L | HIP.L |
| 39,40 | ParaHippocampal_L | PHG.L |
| 41,42 | Amygdala_L | AMYG.L |
| 83,84 | Temporal_Pole_Sup_L | TPOsup.L |
| 87,88 | Temporal_Pole_Mid_L | TPOmid.L |
| Subcortical |  |  |
| 21,22 | Olfactory_L | OLF.L |
| 71,72 | Caudate_L | CAU.L |
| 73,74 | Putamen_L | PUT.L |
| 75,76 | Pallidum_L | PAL.L |
| 77,78 | Thalamus_L | THA.L |
| Occipital |  |  |
| 43,44 | Calcarine_L | CAL.L |
| 45,46 | Cuneus_L | CUN.L |
| 47,48 | Lingual_L | LING.L |
| 49,50 | Occipital_Sup_L | SOG.L |
| 51,52 | Occipital_Mid_L | MOG.L |
| 53,54 | Occipital_Inf_L | IOG.L |
| 55,56 | Fusiform_L | FFG.L |

The regions are listed in terms of a prior AAL atlas (Tzourio-Mazoyer et al., 2002). Odd and even numbers represent brain regions of left and right hemispheres, respectively. Six main groups were derived from (Salvador et al., 2005)

Abbr., abbreviations.

**References**

Salvador, R., Suckling, J., Coleman, M.R., Pickard, J.D., Menon, D., Bullmore, E., 2005. Neurophysiological Architecture of Functional Magnetic Resonance Images of Human Brain. Cerebral Cortex. 15**,** 1332-1342.

Tzourio-Mazoyer, N., Landeau, B., Papathanassiou, D., Crivello, F., Etard, O., Delcrox, N., Mazoyer, B., Joliot, M., 2002. Automated anatomical labeling of activations in SPM using a macroscopic anatomical parcellation of the MNI MRI single-subject brain. NeuroImage. 15**,** 273–289.
